# A novel irrigation device with superior nasal irrigation efficiency to the classic rinse bottle

**DOI:** 10.1186/s40463-022-00575-9

**Published:** 2022-05-03

**Authors:** Dawei Wu, Feifan Chang, Junsheng Hong, Baihan Su, Yongxiang Wei

**Affiliations:** 1 Department of Otolaryngology-Head and Neck Surgery, Peking University Third Hospital, Beijing, People’s Republic of China; 2 Beijing Institute of Heart Lung and Blood Vessel Diseases, Beijing, People’s Republic of China; 3 Department of Otolaryngology, Beijing Anzhen Hospital, Capital Medical University, Beijing, People’s Republic of China; 4 Department of Otorhinolaryngology-Head and Neck Surgery, Capital Institute of Pediatrics, Beijing, People’s Republic of China

**Keywords:** Nasal irrigation, Irrigation efficiency, Removal effect, Nasal cavity, Fluid flow, Head position

## Abstract

**Background:**

The ability of saline irrigation to detach the mucous and the flow-limiting effect of the nasal valve has not been well explored. The objective of this study was to compare the removal efficiency of a novel irrigation device with an extended nozzle versus a classic rinse bottle.

**Methods:**

Transparent casts of the unoperated sinonasal cavity were made by 3D printing. Yogurt was used to simulate mucous. The cast filled with 5 ml yogurt was fixed in six head positions and irrigated with 120 ml, 175 ml, and 240 ml dyed water through the novel device and the rinse bottle. The irrigation efficiency was the ratio of the weight of yogurt washed away divided by the total weight of yogurt.

**Results:**

The irrigation stream of a long nozzle with a side opening was different from the irrigation stream of the outlet within the nasal vestibule. The novel devices presented with continuous water stream directly upwards to the anterior part of the olfactory cleft. Depending on different head positions, it was easy for the novel devices to achieve an irrigation efficiency of 100% when the cast was irrigated with 120 ml or 175 ml water. There was still a tiny amount of yogurt left in the olfactory cleft when the cast was irrigated with 240 ml water under each head position for the rinse bottle. The irrigation efficiency was volume-dependent, and the average irrigation efficiency of the rinse bottle at 240 ml only reached 69.1%.

**Conclusions:**

The novel irrigation device presented with superior nasal irrigation efficiency to the classic rinse bottle. A continuous water stream directly upwards to the anterior part of the olfactory cleft combined with an extended nozzle overcoming the flow-limiting effect of the nasal valve promotes nasal irrigation efficiency.

**Graphical Abstract:**

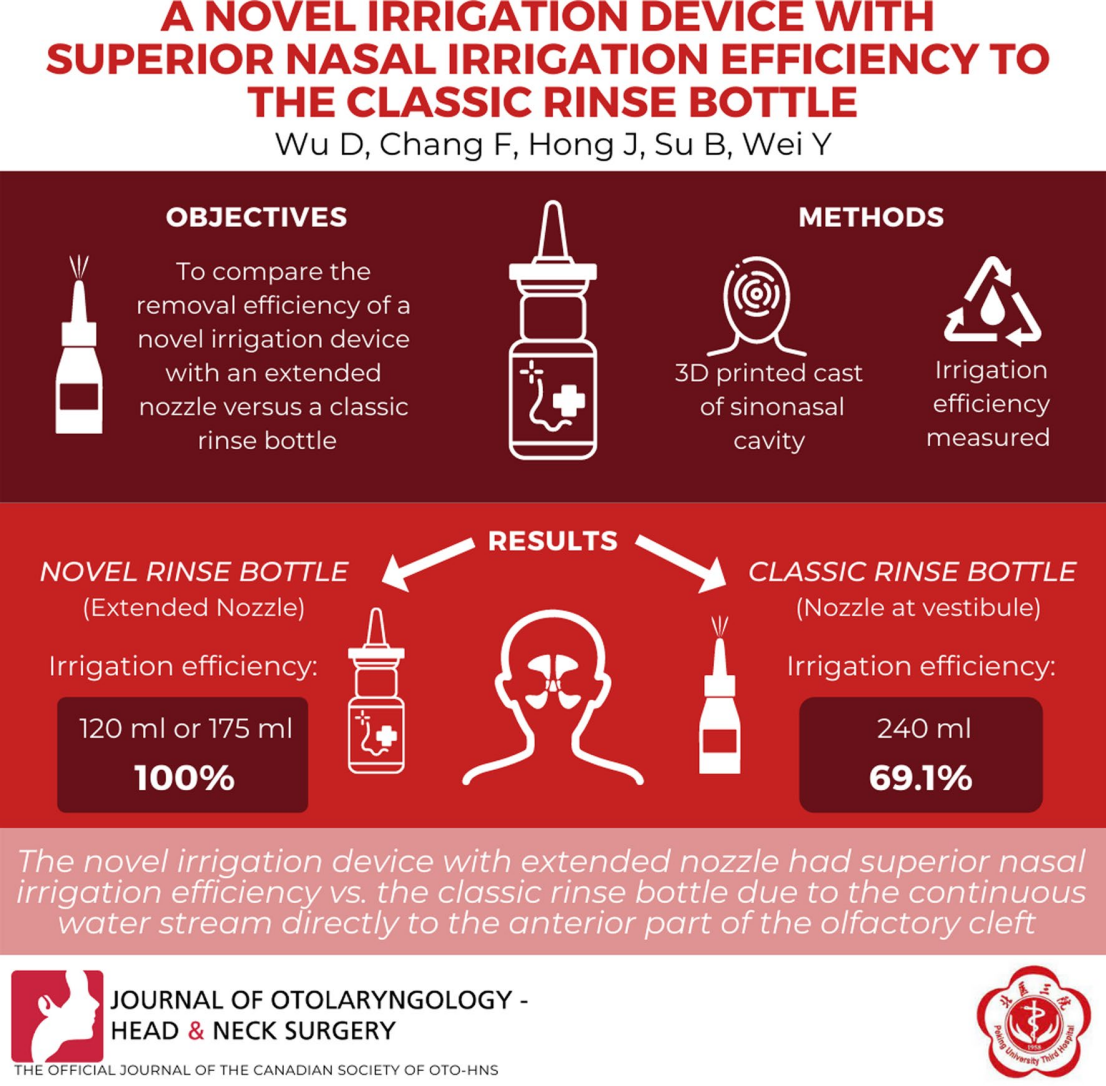

## Introduction

Nasal irrigation has been recommended as an important adjunct therapy for sinonasal diseases before and after surgery [1, 2]. Nasal irrigation could significantly relieve the sinonasal symptoms and improve the quality of life among patients with sinonasal disorders mainly through a direct mechanical clearing of the secretion, nasal crusts, infective pathogens, and inflammatory cytokines’ load within the sinonasal cavity [3–5]. There is accumulating evidence showing that various irrigating devices, including a squeeze bottle and pulse irrigation device, presented with different efficacy regarding the fluid coverage area of the nasal cavity, sinus penetration, and retained amount of fluid [6–8].

Furthermore, many factors, including the head position, delivery volume, and the degree of surgery, have been identified which significantly influenced the irrigation efficacy [9–12]. Moreover, the efficacy based on the degree of the staining, calculation of the stained area, percentage of the fill of the sinus, and retained amount of irrigants has been evaluated in nasal cast models, healthy participants, and cadaveric dissections using dyed or fluorescein-labeled irrigants, computational fluid dynamics (CFD) simulation, or imaging methods [6, 13–17]. These studies mainly focused on the effect of different irrigation techniques on the size and area of the fluid coverage and retained amount of fluid within the sinonasal cavity. It has been proposed that the contact of water flow with the nasal well, how long the contact time, and flow momentum (a combination of velocity and liquid volume) are critical factors determining the ability to detach the secretion or crusts [18, 19]. There was no study to explore the removal efficiency of mucous after irrigation. Establishing quantifiable rinsing effectiveness metrics would further facilitate the comparison of various irrigation devices in the removal efficiency and then help optimize nasal irrigation devices.

The design and characteristics of the device itself significantly influenced the distribution patterns of fluid flow within the sinonasal cavity [7, 20]. Specifically, an outlet that can seal the nostril and be inserted into the nasal vestibular made it possible to irrigate the whole nasal cavity and paranasal sinuses [21]. In addition to the device itself, the nasal valve located 2 to 3 cm from the anterior nostril is the narrowest site within the nasal cavity and acts as a flow-limiting region [22]. However, most commercially available irrigation devices have short nozzles which can only be inserted into the nasal vestibular. Previous studies demonstrated that the nasal valve dimensions significantly decreased the drug delivery efficiency of the nasal spray to the remote upper and posterior nasal cavity, leading to a significant fraction of drug deposited in the anterior region to the nasal valve [23–25]. Based on the above findings, we speculated that compressible nasal irrigation devices with a nozzle with a penetration distance beyond the nasal valve would overcome the weakening effect to the flow momentum due to the fluid friction or turbulence within the nasal vestibular.

This study aimed to compare the novel irrigation device versus a classic squeeze bottle concerning the distribution patterns of fluid flow and irrigation efficiency for the whole nasal cavity. Yogurt was used to simulate mucous, and a procedure for quantifying the irrigation efficiency was built based on the ratio of the weight of yogurt washed away divided by the total weight of yogurt.

## Materials and methods

### Development of the left sinonasal model and an adjustable apparatus for fixing the model

In order to visualize the rinsing process of the nasal cavity from the lateral view of the side of the nasal septum, left sinonasal models were prepared using a 3D printer (Stratasys J750™, Digital Anatomy™), and the sinonasal 3D surface data was converted from the high-resolution nasal sinus CT scans of a healthy 35-year-old Asian male (resolution, 512 × 512; layer thickness, 1 mm; window width, 2000 HU; window position, 400 HU). The ultra-realistic anatomical simulation was printed with composite materials. Three turbinates (superior, middle, and inferior), the surface of the left nasal cavity, the nasal vestibule, and the nasal septum were printed using rubber-like translucent material Agilus30 ™ while the rest of the 3D model was printed with rigid transparent material VeroClear ™. The nasal septum could be tightly and easily combined with the left sinonasal cavity. The connection between the nasal septum and the left sinonasal cavity was sealed with soft glue to maintain airtight except for the nostril and tracheal opening. A validated apparatus for fixing the 3D nose model has been developed in our previous study [26]. This platform was designed to provide coronal, sagittal, and horizontal planes, keeping the cast in a specific head position. A phone (iPhone X; Apple, Cupertino, CA, US) was placed in front of the apparatus at a fixed distance to record the irrigation.

### Irrigation experiment procedures and calculation of the nasal irrigation efficiency

**Fig. 1:**
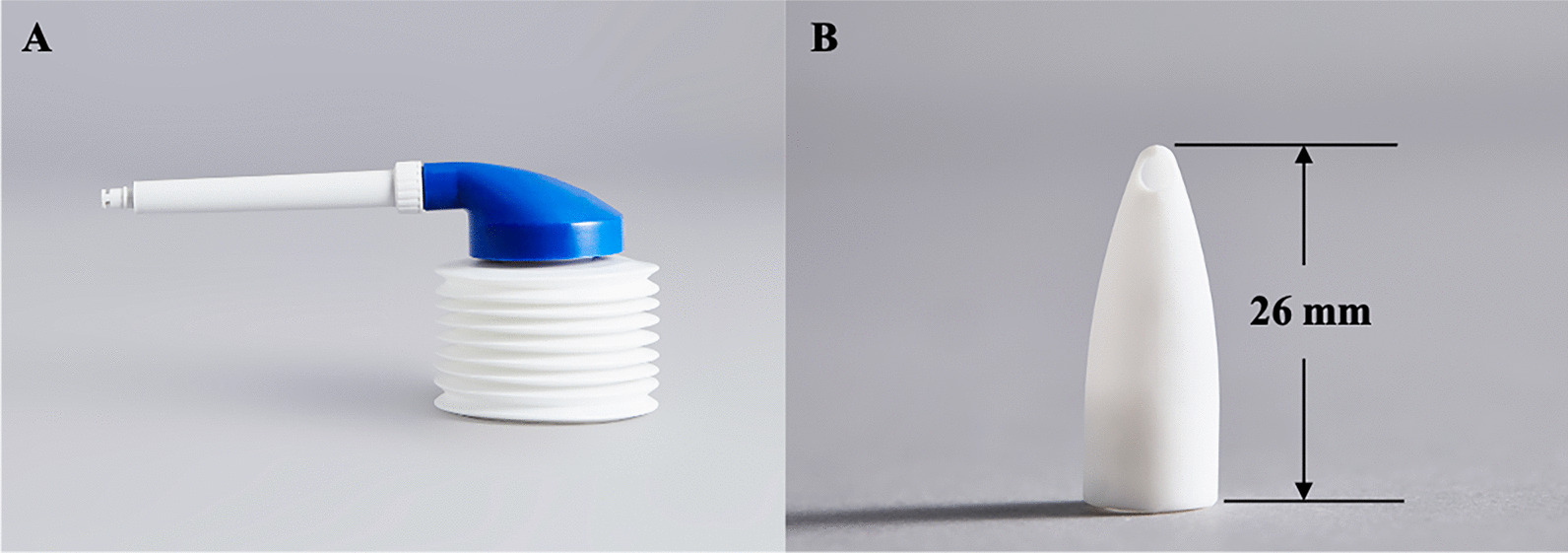
A novel irrigation device. **A** The accordion-like squeeze bottle connected to a rigid plastic catheter; **B** A 26 mm silicone nozzle with a side opening on the tip and plastic catheter can be tightly inserted into the nozzle

A novel irrigation device (lean Pharmaceutical Technology Co., Ltd., Beijing, China), including an accordion-like squeeze rinse bottle, rigid catheter, and rinse nozzle with side opening, was utilized to rinse the model (Fig. 1). We chose a widely used Sinus Rinse bottle (NeilMed, Inc., Santa Rosa, CA, US) to compare the irrigation effect with the novel irrigation device. Distilled water dyed with blue food coloring was utilized to show the fluid flow distribution within the nasal cavity. A total of six head positions were applied: (1) tilt 10 degrees forward (T10F); (2) tilt 45 degrees forward (T45F); (3) tilt 60 degrees forward (T60F); (4) tilt 10 degrees forward and 30 degrees to the right (T10F-30R); (5) tilt 45 degrees forward and 30 degrees to the right (T45F-30R); (6) tilt 60 degrees forward and 30 degrees to the right (T60F-30R). Three different volumes, including 120 ml, 175 ml, and 240 ml, were tested for each of the different head positions. We took the following three-step procedure to generate the irrigation efficiency [26].

1. The cast was tightly fixed in one of the six head positions. The nozzle of the nasal irrigation device was engaged in the nostril with a fixed bottle position and squeezed with constant pressure until empty for the bottle. Similarly, the nozzle of the rinse bottle was engaged in the nostril and squeezed with constant pressure, then released, and pressure reapplied. The cast was rinsed twice with dyed water at a constant speed and then stood still for 5 min. The cast was gently removed from the apparatus, and the wet weight of the cast was recorded (W1).
2. The nasal septum was gently separated from the cast of the left sinonasal model**,** and 5 ml yogurt with a whey protein concentration of 8% was distributed with a syringe for even application covering around the superior, middle, and inferior turbinate. The nasal septum was combined again with the left sinonasal model. The weight of the cast filled with 5 ml yogurt was re-recorded (W2).
3. The cast was fixed in the same head position, and the left sinonasal cavity was rinsed once with the same volume of the dyed water. After standing for 5 min, the cast was gently removed from the apparatus. Finally, the weight of the cast with the remaining yogurt was weighed (W3). The irrigation efficiency (ΔW) was calculated based on the weight difference before and after flushing the model filled with yogurt. The irrigation efficiency was the ratio of the weight of yogurt washed away (W2-W3) divided by the total weight of yogurt (W2-W1). The equation of irrigation efficiency was: ΔW = (W2-W3)/(W2-W1).

The left sinonasal model was washed with distilled water and air-dried before the next irrigation experiment to remove previously administered yogurt and water completely. For each volume (120 ml, 175 ml, and 240 ml) in a specific head position, we have repeated the procedure three times to obtain the average irrigation efficiency. If one of the water volumes is enough to flush out all the simulated nasal mucous, we will stop at that water volume. In order to minimize the testing bias, a single investigator who performed the irrigation would repeat many times to obtain a uniform squeeze pressure and stable positions of the irrigation devices.

### Statistical analysis

The irrigation efficiency was presented as mean ± standard deviation (SD). The 2-sample t-test was applied to analyze the difference in the irrigation efficiency between two volume groups. Significant differences among multiple groups were determined using a Kruskal–Wallis test. A p-value of less than 0.01 was considered statistically significant. Statistical analyses were performed using Prism 8 (GraphPad Software) and SPSS statistical software, version 23.0 (IBM Corp., Armonk, NY).

## Results

### Distribution patterns of fluid flow within the nasal cavity

We analyzed each video frame and summarized the patterns of the dynamic fluid flow over time. It took about 7 s, 10 s, and 14 s for the accordion-like bottle of the novel device to finish the irrigation volume of 120 ml, 175 ml, and 240 ml, respectively. The rinse bottle was consistently squeezed and then released. There was no flushing effect during the release of the rinse bottle. We only calculated the effective squeeze time, and it took about 8 s, 13 s, and 15 s for the rinse bottle to finish the irrigation volume of 120 ml, 175 ml, and 240 ml, respectively.

**Fig. 2:**
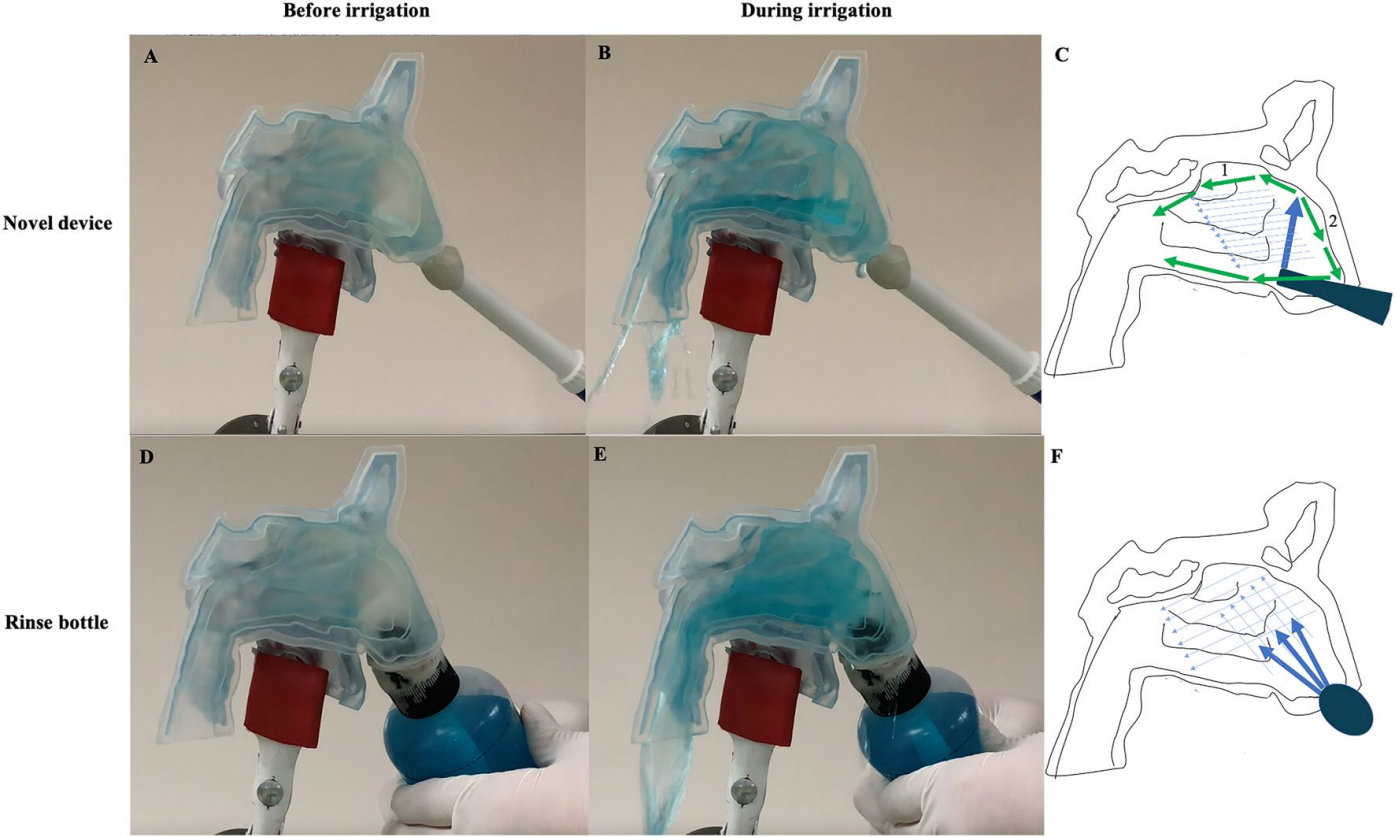
Distribution patterns of fluid flow during the squeeze with the novel irrigation device and the rinse bottle (head tilt 10 degrees forward with leaning 30 degrees to the right). **A**, **B** The status of the nasal cavity before and during irrigation with the novel device; **C** Schematic diagram of the main water flow during irrigation with the novel device; **D**, **E** The status of the nasal cavity before and during irrigation with the rinse bottle; **F** Schematic diagram of the main water flow during irrigation with the rinse bottle

The water flow and nasal surface coverage within the nasal cavity varied due to the different head positions. In order to present the typical water flow distribution difference between the novel irrigation device and the rinse bottle, we described the fluid flow when the model was fixed in the T10-30R position (Fig. 2). For the novel irrigation device, the water was constantly sprayed to the nasofrontal beak located within the anterior aspect of the olfactory cleft, and then the direction of the flow of water was mainly divided into two separate directions (Fig. 2A, B). One water flow continued moving from front to back along the olfactory cleft. Another water flow moved along the dorsum nasi and was folded back to the bottom of the nasal cavity (Fig. 2C). Due to the flushing pressure and gravity, the whole water filled the whole nasal cavity and converged toward the posterior nostril. The rinse bottle was squeezed with constant pressure until the bottle was fully compressed, and the rinse bottle was immediately released until the shape of the bottle was fully recovered (Fig. 2D, E). The water was sprayed to the whole nasal cavity during the squeeze, and the level of fluid flow dropped to the bottom of the nasal cavity during the release (Fig. 2F).

### The removal effect and irrigation efficiency of the novel irrigation device

**Fig. 3:**
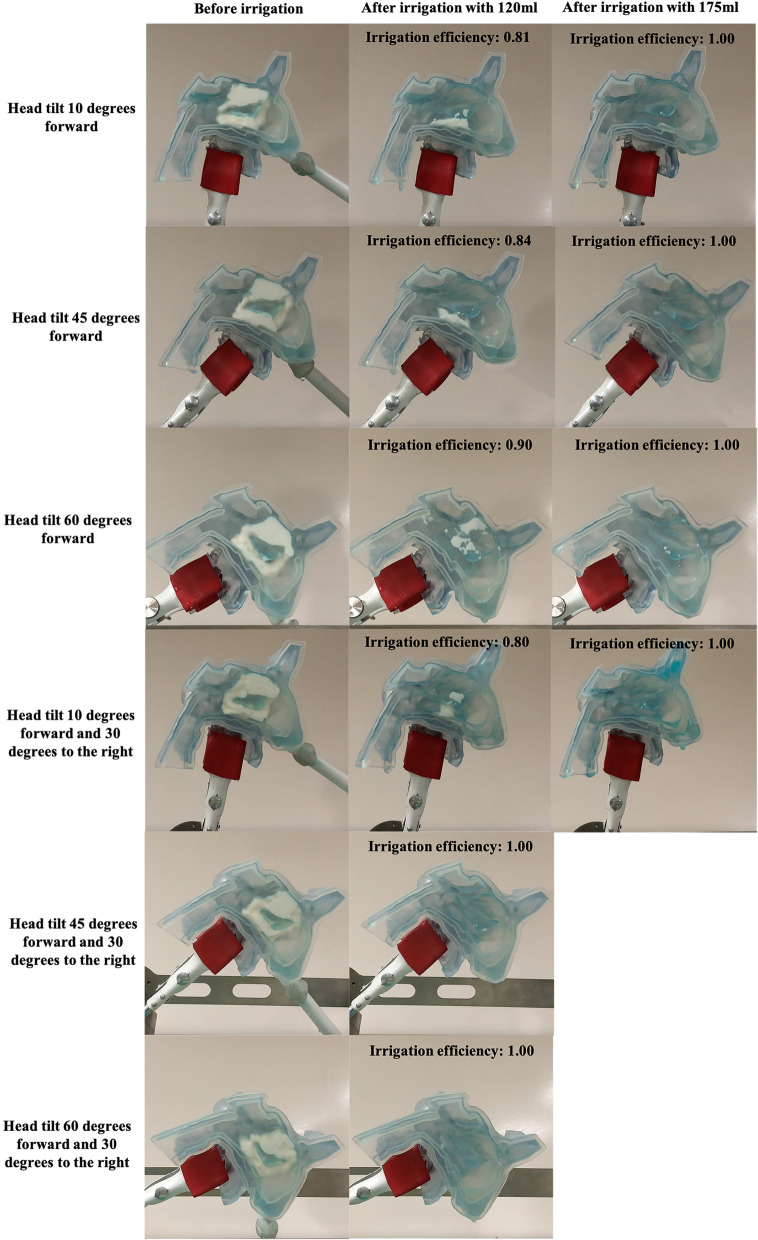
The novel device's removal effect and irrigation efficiency under six different head positions. Each cast was filled with 5 ml simulated nasal mucous. Under the T10F, T45F, T60F, or T10F-30R position, the simulated mucous around the superior and middle turbinate was preferentially removed, and the irrigation efficiency of the novel device achieved 100% at the 175 ml water. The irrigation efficiency of novel devices achieved 100% at the 120 ml water when the cast was fixed in the T45F-30R or T60F-30R position. T10F, tilt 10 degrees forward; T45F, tilt 45 degrees forward; T60F, tilt 60 degrees forward; T10F-30R, tilt 10 degrees forward and 30 degrees to the right; T45F-30R, tilt 45 degrees forward and 30 degrees to the right; T60F-30R, tilt 60 degrees forward and 30 degrees to the right

When the cast was fixed in T10F, T45F, T60F, or T10F-30R position and was irrigated with 120 ml water, the remaining yogurt was either located around the inferior turbinate (T10F, T45F, T10F-30R) or the posterior part of the olfactory cleft (T60F) (Fig. 3 and Table 1). The irrigation efficiency of the novel irrigation device under the four positions ranged from 80.1 to 89.8%, with an average irrigation efficiency of 83.6%. Furthermore, no yogurt was left when the cast was irrigated with higher water volume (175 ml) in the above four head positions. The irrigation efficiency with 175 ml water was significantly higher than with 120 ml water for each of the four head positions, including T10F, T45F, T60F, and T10F-30R (all *p* < 0.0001). Interestingly, the irrigation efficiency reached 100% when the cast was fixed in the T45F-30R or T60F-30R position and irrigated with 120 ml water (Fig. 3 and Table 1).

**Table 1:** Irrigation efficiency of the novel device

Characteristic	120 ml	175 ml	*P* value between two volumes
Irrigation efficiency in T10F (%), mean (SD)	80.7 (0.8)	100%	< 0.0001
Irrigation efficiency in T45F (%), mean (SD)	83.7 (1.0)	100%	< 0.0001
Irrigation efficiency in T60F (%), mean (SD)	89.8 (1.3)	100%	< 0.0001
Irrigation efficiency in T10F-30R (%), mean (SD)	80.1 (0.4)	100%	< 0.0001
Irrigation efficiency in T45F-30R (%), mean (SD)	100%	–	–
Irrigation efficiency in T60F-30R (%), mean (SD)	100%	–	–

T10F, tilt 10 degrees forward; T45F, tilt 45 degrees forward; T60F, tilt 60 degrees forward; T10F-30R, tilt 10 degrees forward and 30 degrees to the right; T45F-30R, tilt 45 degrees forward and 30 degrees to the right; T60F-30R, tilt 60 degrees forward and 30 degrees to the right; SD, standard deviation

### The removal effect and irrigation efficiency of the rinse bottle

**Fig. 4:**
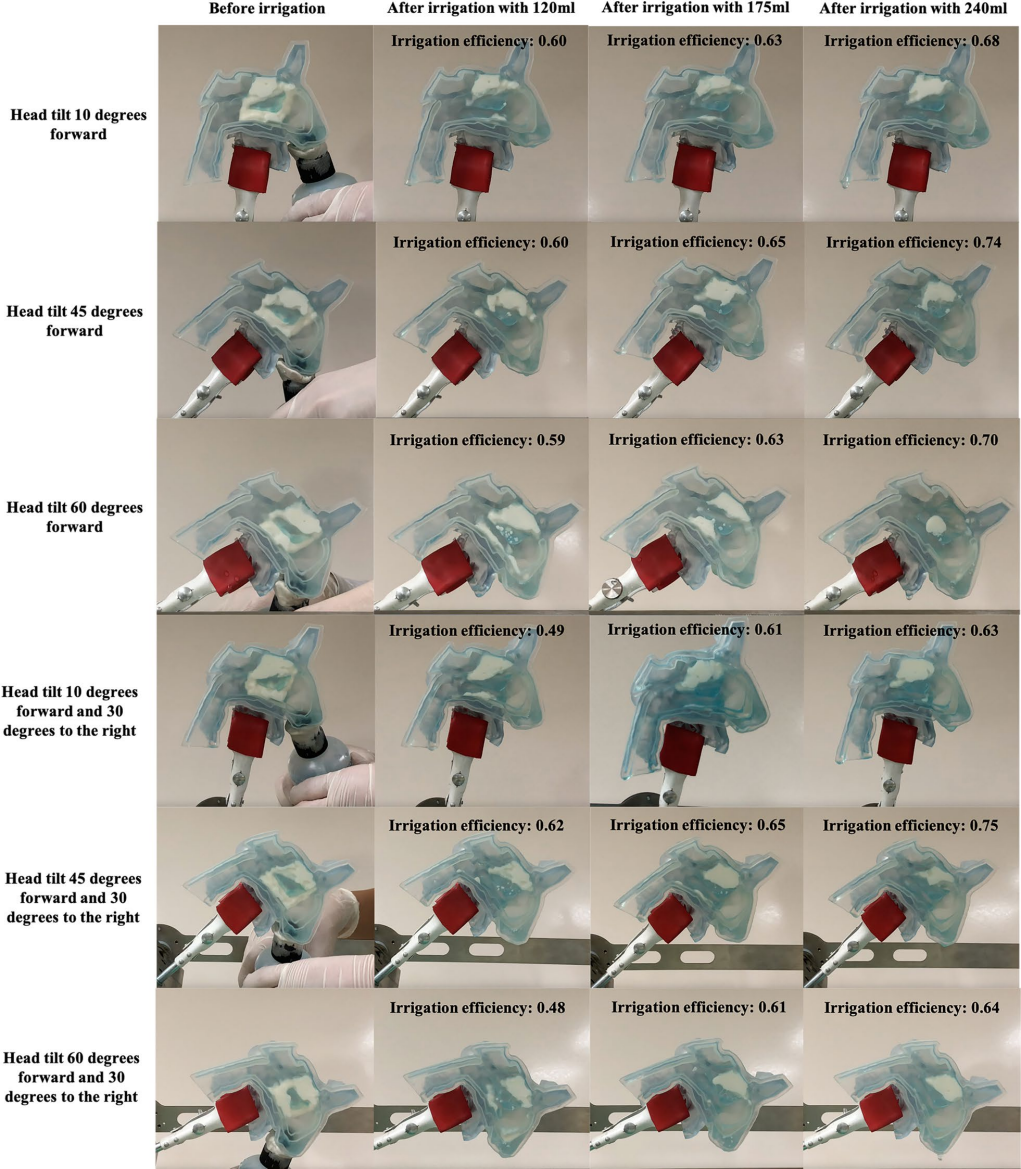
The rinse bottle's removal effect and irrigation efficiency under six different head positions. Each cast was filled with 5 ml simulated nasal mucous. The irrigation efficiency was volume-dependent, and the head positions significantly influenced the irrigation efficiency. A tiny amount of yogurt remained only in the olfactory cleft after irrigation with 240 ml water

We next analyzed the remaining yogurt distribution patterns. For each of the six head positions (T10F, T45F, T60F, T10F-30R, T45F-30R, and T60F-30R), the remaining yogurt was mainly located around the superior turbinate and the inferior turbinate when the cast was irrigated with 120 ml water (Fig. 4 and Table 2). The remaining yogurt was less when the cast was irrigated with higher water volume for each of the six head positions, with the lowest amount of yogurt under 240 ml water. The irrigation efficiency was significantly different among the 120 ml, 175 ml, and 240 ml groups for each head position (all *p* < 0.0001) (Fig. 4 and Table 2). When the cast was fixed in each of the six head positions and irrigated with 240 ml water, only a tiny amount of yogurt remained in the olfactory cleft, and the irrigation efficiency of the rinse bottle ranged from 66.3% to 75.4% with an average irrigation efficiency of 69.1%.

**Table 2:** Irrigation efficiency of the rinse bottle

Characteristic	120 ml	175 ml	240 ml	*P* value among different volumes
Irrigation efficiency in T10F (%), mean (SD)	59.8 (1.0)	63.0 (0.7)	68.2 (1.0)	< 0.0001
Irrigation efficiency in T45F (%), mean (SD)	60.1 (0.8)	65.3 (0.8)	73.8 (0.6)	< 0.0001
Irrigation efficiency in T60F (%), mean (SD)	59.2 (0.8)	62.8 (0.9)	70.2 (1.3)	< 0.0001
Irrigation efficiency in T10F-30R (%), mean (SD)	49.0 (0.9)	60.5 (0.8)	62.6 (1.0)	< 0.0001
Irrigation efficiency in T45F-30R (%), mean (SD)	62.3 (0.9)	65.1 (0.6)	75.4 (1.0)	< 0.0001
Irrigation efficiency in T60F-30R (%), mean (SD)	48.4 (0.9)	61.1 (1.3)	64.3 (0.6)	< 0.0001

T10F, tilt 10 degrees forward; T45F, tilt 45 degrees forward; T60F, tilt 60 degrees forward; T10F-30R, tilt 10 degrees forward and 30 degrees to the right; T45F-30R, tilt 45 degrees forward and 30 degrees to the right; T60F-30R, tilt 60 degrees forward and 30 degrees to the right; SD, standard deviation

## Discussion

Achieving increased liquid surface coverage and high irrigation penetration within the sinonasal cavity has been the primary goal of nasal irrigation devices. Previous studies have emphasized the importance of fluid coverage and the deposition of irrigants within the sinonasal cavity, and quantifiable metrics about these two aspects have been developed [6, 13–17]. It has been observed that large-volume irrigation (> 100 ml) with proper head position would cover all zones of the postoperative sinonasal cavity and promote drug delivery [6, 10, 19, 27]. However, it is not sufficient for an irrigation device to fill the sinonasal cavity, and the water flow should remove all the debris. Strong shear stress and prolonged contact time of the irrigation stream were essential to remove the viscous nasal mucous and the sticky debris [19]. The anterior triangular segment of the nasal anatomy called the nasal valve is the main flow-limiting segment, which modifies the rate and direction of the airflow [28, 29]. However, the flow-limiting effect of the nasal valve during the irrigation was not well known.

This study compared a novel irrigation device with a classic rinse bottle and found that the irrigation stream of a nozzle with a penetration distance beyond the nasal valve was different from the irrigation stream of the outlet within the nasal vestibular. The novel irrigation device sprayed a continuous irrigation stream directed upwards to the anterior aspect of the olfactory cleft. The stream was then divided into two major directions, which was sufficient to cover all the nasal cavity. As expected, the water of the rinse bottle covered the whole nasal cavity rapidly during the squeeze, which was consistent with the results of the previous in vitro studies [19, 21]. This is the first study exploring the distribution pattern of fluid flow irrigated from a long nozzle bypassing the nasal valve area.

The distinct distribution patterns of the fluid flow within the nasal cavity between these two devices showed a different capacity to flush out the nasal secretions. These observations led us to further quantitatively analyze the removal efficiency of mucous. With the development of 3D printing technology and improved material performance, a nasal cast that stimulates the actual sinonasal cavity can be well prepared [30]. For the first time, we utilized yogurt as the simulated nasal mucous. We found that the nasal irrigation efficiency of these two irrigation devices was volume-dependent, and the head positions significantly influenced the irrigation efficiency. For novel devices, the simulated mucous around the superior and middle turbinate was preferentially removed when the cast was fixed in the T10F, T45F, T60F or T10F-30R position and irrigated with 120 ml water. The average irrigation efficiency of novel devices under the above four positions was as high as 83.6%. The remaining yogurt located around the inferior turbinate or the posterior part of the olfactory cleft was removed totally when the flushing volume was increased to 175 ml. 100% of the simulated mucous was flushed away when the cast was fixed in the T45F-30R, or T60F-30R position and irrigated with 120 ml water. As for the classic rinse bottle, the patterns of the remaining yogurt were different from that of the novel device. For each of the six head positions (T10F, T45F, T60F, T10F-30R, T45F-30R, and T60F-30R), the simulated mucous around the middle and inferior turbinate was preferentially removed when the cast was irrigated with 120 ml water. Moreover, a tiny amount of yogurt remained only in the olfactory cleft after irrigation with 240 ml water with an average irrigation efficiency of 69.1%. Several mechanisms may explain the distinct removal effect of these two devices. Compared to the classic rinse bottle, the novel device has the following three essential characteristics: (1) one full compression with steadier stream and longer contact time, (2) a long nozzle overcoming the weakening effect of the nasal valve to the flow momentum, and (3) a side opening allowing for direct stream upwards to the anterior part of the olfactory cleft. To our knowledge, this is the first in vitro study exploring the removal effect of nasal irrigation, and we propose a novel device with vital features favoring high irrigation efficiency.

Although head tilt positions with large angle (e.g., nose-to-floor or nose-to-ceiling head position) was optimal to irrigate during the irrigation, it may be difficult for patients of different age to adopt and maintain the position [31, 32]. Therefore, relatively low water volume, high irrigation efficiency, and a comfortable head position to follow were desirable strategies for choosing the nasal irrigation technique. Based on the tested volumes and head positions in the present study, the novel devices under 120 ml water and the T45F-30R position achieved the highest irrigation efficiency of 100%. In contrast, the rinse bottle under the 240 ml water and T45F-30R position performed best regarding the irrigation efficiency compared with other conditions. However, the highest irrigation efficiency of the rinse bottle was only 75.4%, and there was still a tiny amount of the simulated mucous in the olfactory cleft, reflecting the inherent defects of the rinse bottle. Although the effective squeeze time of the novel device and the rinse bottle was similar during the irrigation with the same water volume, the novel device maintained a constant water flow while the rinse bottle showed interruption and backflow of water flow during the release of the bottle. This may explain, at least partly, the highly efficient flushing of the novel irrigation device.

There are several limitations to our study. First, this study focused on evaluating the fluid flow and the irrigation efficiency within the unoperated nasal cavity of a nose model by 3D printing. The sinus penetration and the removal effect of the irrigation stream of the novel device on the paranasal sinuses should be further explored. Second, this is an in vitro study, and the novel irrigation device provided a mechanical shear force that was sufficient to clean the stimulated mucous. An in vivo study in patients with various sinonasal disorders should be conducted to determine its clinical significance.

## Conclusion

Our study demonstrated that the novel irrigation device presented with superior irrigation efficiency to the classic rinse bottle. Through building a procedure to evaluate the distribution of fluid flow and irrigation efficiency within the nasal cavity, we put forward a series of critical structural and dynamical features for optimizing nasal irrigation devices, including a continuous water stream directly upwards to the anterior part of the olfactory cleft and a long nozzle overcoming the flow-limiting effect of the nasal valve. Further studies are needed to assess treatment effect, patient comfort, and safety in the clinical setting [33].

## Data Availability

All data gathered for the systematic review was gathered from articles cited in the paper and listed in the reference section.

## Abbreviations

CFD: Computational fluid dynamics
SD: Standard deviation
